# Sinistral Portal Hypertension after Pancreaticoduodenectomy with Splenic Vein Resection: Pathogenesis and Its Prevention

**DOI:** 10.3390/cancers13215334

**Published:** 2021-10-24

**Authors:** Yoshihiro Ono, Yosuke Inoue, Tomotaka Kato, Kiyoshi Matsueda, Atsushi Oba, Takafumi Sato, Hiromichi Ito, Akio Saiura, Yu Takahashi

**Affiliations:** 1 Division of Hepatobiliary and Pancreatic Surgery, Cancer Institute Hospital, Japanese Foundation for Cancer Research, Tokyo 135-8550, Japan; yoshihiro.ono@jfcr.or.jp (Y.O.); tomotaka.kato@jfcr.or.jp (T.K.); atsushi.oba@jfcr.or.jp (A.O.); takafumi.sato@jfcr.or.jp (T.S.); hiromichi.ito@jfcr.or.jp (H.I.); yu.takahashi@jfcr.or.jp (Y.T.); 2 Department of Diagnostic Imaging, Cancer Institute Hospital, Japanese Foundation for Cancer Research, Tokyo 135-8550, Japan; kiyochi.matsueda@jfcr.or.jp; 3 Department of Hepatobiliary-Pancreatic Surgery, School of Medicine, Juntendo University, Tokyo 113-0033, Japan; a-saiura@juntendo.ac.jp

**Keywords:** sinistral portal hypertension, pancreatic cancer, pancreaticoduodenectomy, splenic vein ligation/resection, gastrointestinal varices, gastrointestinal bleeding

## Abstract

**Simple Summary:**

Extensive portal vein (PV) resection, including porto-mesenterico-splenic confluence (PMSC) during pancreaticoduodenectomy (PD) may sometimes be necessary for pancreatic head cancer, if the tumor is close to the portal venous system. However, as a late-onset postoperative complication, this extensive PV resection may result in sinistral portal hypertension (SPH) and cause variceal bleeding due to congested venous flow from the spleen. Since the prognosis of patients with pancreatic cancer has improved, owing to the development of chemotherapy and surgical techniques, SPH is no longer a negligible matter in the field of pancreatic cancer surgery. This review clarifies the pathogenesis and frequency of SPH after PD with PMSC resection and discusses its prediction and prevention.

**Abstract:**

To achieve curative resection for pancreatic cancer during pancreaticoduodenectomy (PD), extensive portal vein (PV) resection, including porto-mesenterico-splenic confluence (PMSC), may sometimes be necessary if the tumor is close to the portal venous system. Recently, this extended resection has been widely accepted in high-volume centers for pancreatic resection due to its favorable outcomes compared with non-operative treatment. However, in patients with long-term survival, sinistral portal hypertension (SPH) occurs as a late-onset postoperative complication. These patients present gastrointestinal varices due to congested venous flow from the spleen, which may cause critical variceal bleeding. Since the prognosis of patients with pancreatic cancer has improved, owing to the development of chemotherapy and surgical techniques, SPH is no longer a negligible matter in the field of pancreatic cancer surgery. This review clarifies the pathogenesis and frequency of SPH after PD through PMSC resection and discusses its prediction and prevention.

## 1. Introduction

Sinistral (left-sided) portal hypertension (SPH) was originally reported by Turrill et al. in 1969 as gastroesophageal variceal bleeding resulting from the splenic vein (SV) occlusion [1]. This symptom has been well reported since the 1900s, and a review article in 1970 summarized the etiology of isolated SV occlusion as follows: neoplasia, pancreatitis, trauma, pseudocyst, infection, miscellaneous, and unknown [2]. Thereafter, Evans [3] defined “Sinistral (left-sided) portal hypertension” as a clinical syndrome of SV thrombosis caused by pancreatic pathology which manifests as bleeding in the gastric varices in patients with a patent portal vein (PV) and normal hepatic function.

The same symptoms were observed in patients who underwent pancreaticoduodenectomy (PD) with SV resection. Fortner et al. reported symptoms such as hemorrhagic stomach, an enlargement of the spleen, or spontaneous splenic rupture during regional subtotal pancreatectomy with SV ligation as a result of venous congestion [4]. They also commented that such congestion was a rare occurrence, although a splenectomy was necessary during surgery in some cases. Since then, several reports have been published regarding the occurrence of gastrointestinal varices and bleeding after PD with porto-mesenterico-splenic confluence (PMSC) resection [5,6,7,8,9]. However, due to the poor survival rate of patients with pancreatic ductal adenocarcinoma (PDAC) who underwent PV resection, reports of coherent cases are limited. In recent years, the prognosis of PDAC has gradually improved due to the progress of multidisciplinary therapies, including chemotherapy and radiotherapy, and accordingly, the number of reports on SPH has been increasing over the past decade.

The definition of SPH after PD varies among reports. The occurrence of bleeding from varicose veins after SV ligation/resection without liver disease or PV stenosis/occlusion would correspond to the original definition [3,10], however, the incidence of varicose veins, the enlargement of the spleen, thrombocytopenia, and persistent abdominal pain were also listed in the previous study as defining features of SPH [11,12,13,14,15,16,17,18,19,20]. It should be noted that the direct cause of gastrointestinal bleeding is varicose vein formation and patients with varicose veins are at risk of gastrointestinal bleeding in the future. Postoperative spleen hypertrophy, thrombocytopenia, and abdominal pain may result from the high SV pressure or splenomegaly, but these could also be induced by tumor recurrence, PV stenosis, liver disease, chemotherapy, or various drugs.

Cases without SV resection were previously compared to those with SV resection, and SV resection was proven to increase variceal formation or variceal bleeding [13,21]. However, it can be difficult to preserve SV during PD if the tumor is located close to the PMSC or if tumor invasion is suspected; therefore, a thorough understanding of SPH is necessary for pancreatic surgeons. In this review, we summarize the previous studies regarding SPH after PD with PMSC resection, mainly focusing on the incidence of varicose vein formation and gastrointestinal bleeding, and discuss the pathogenesis and frequency of SPH, and its prevention and prediction.

## 2. Pathogenesis of SPH

To investigate the pathogenesis of SPH following PD, it is necessary to understand the drainage routes from the spleen. There are two distinct pathways for these drainage routes: systemic circulation and PV circulation. The former is a spontaneous splenorenal shunt. This physiological collateral is usually evident postoperatively and occurs in approximately 10% of patients with SV ligation [11]. Spontaneous spleno-adreno-renal shunt and other collateral routes draining into systemic circulation via the retroperitoneal venous system or esophageal submucosa are rare but may develop after PD with PMSC resection [22].

**Figure 1:**
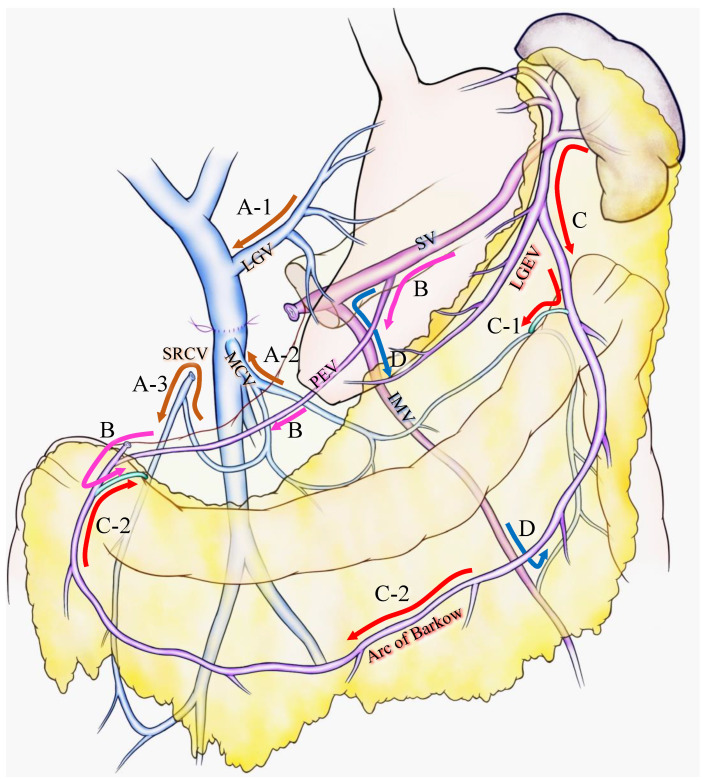
Various venous flow form spleen after portal-superior mesenteric vein confluence resection. Brown arrows indicate the critical veins: (**A-1**) LGV, (**A-2**) MCV, (**A-3**) and SRCV arcade. (**B**) Pink arrows indicate the route from the SV to the PEV and the colonic marginal vein. (**C**) Red arrows indicate the route from the SV to the LGEV and the arc of Barkow; (**C-1**) the connection between the arc of Barkow and the colonic marginal vein at the left side of the transverse colon; (**C-2**) the connection between the arc of Barkow and the colonic marginal vein at the right side of the transverse colon. (**D**) Blue arrows indicate the route from the SV to the IMV and colonic marginal vein. IMV: inferior mesenteric vein; LEGV: left gastric epiploic vein; LGV: left gastric vein; MCV: middle colic vein; PEV: posterior epiploic vein; SV: splenic vein; SRCV: superior right colic vein.

For the latter case, Strasberg et al. introduced two major pathways: the superior route and the inferior route [10,15]. They claimed that the superior route passed from the SV around and through the stomach to enter the PV via the left gastric vein. This route has been previously reported as the classical SPH venous pathway [2,23] and was also stressed as an important SV drainage route to prevent SPH after PD with PMSC resection [14] (Figure 1A-1). The inferior route passed from the SV through the root of the mesentery (the posterior epiploic vein) (Figure 1B), the omental arcade (arc of Barlow), or the colonic vein to the superior mesenteric vein (SMV) (Figure 1C). Strasberg et al. also emphasized the importance of a longer length of residual SV to preserve the posterior epiploic vein or the left gastric epiploic vein, which sometimes flow into the SV in the pancreas and develop as a collateral vein.

**Figure 2:**
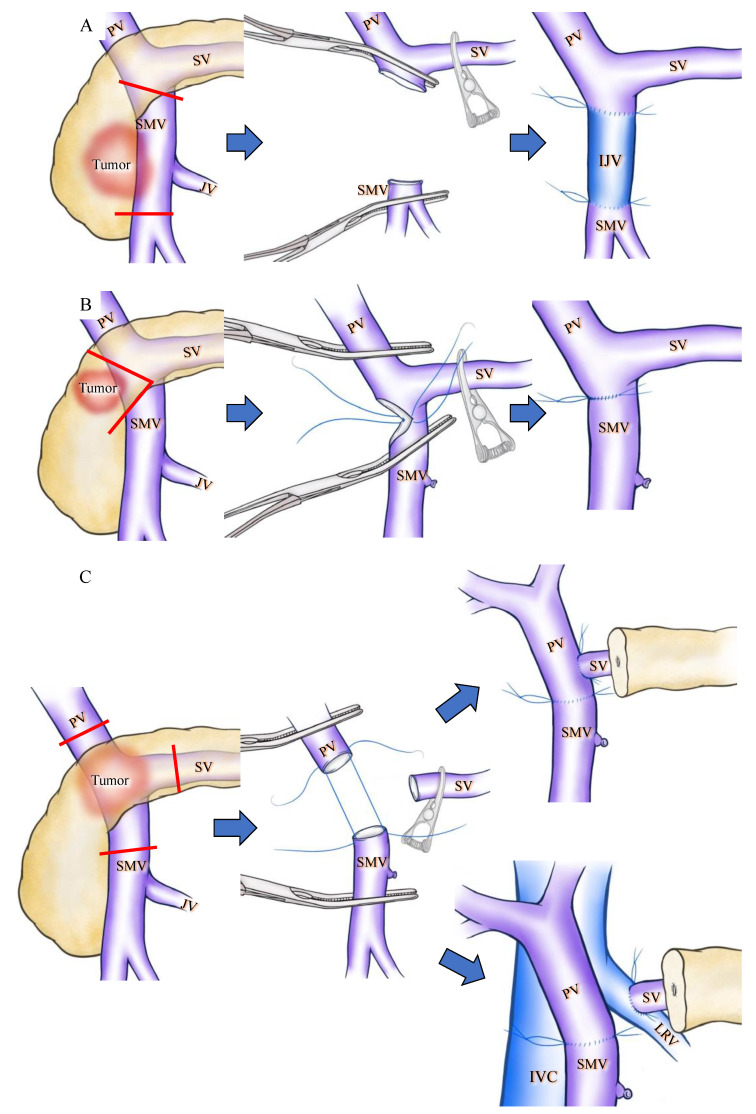
Schematic drawings before and after superior mesenteric vein (SMV) or splenic vein (SV) reconstruction during pan-creaticoduodenectomy (PD). Red lines indicate cut lines of PV, SMV and SV. (**A**) Wide resection of the SMV under SV confluence was performed. IJV was used as the interposition graft. (**B**) Wedge resection of the SMV was performed, and the SMV was reconstructed by direct running suture in a transverse fashion. (**C**) The PV, SMV, and SV were cut as close to the specimen as possible, and SV-PV anastomosis (upper side) and SV-LRV anastomosis (lower side) were performed. IJV: internal jugular vein; IVC: inferior vena caca; LRV: left renal vein; PV: portal vein; SMV: superior mesenteric vein; SV: splenic vein.

Our group has confirmed that these superior and inferior routes [11,17] reported a high incidence (62.8%) of colonic varices, if both the left gastric vein (LGV) and middle colic vein (MCV) were sacrificed during PD with SV ligation [17]. We emphasized the importance of the superior right colic vein (SRCV) arcade to prevent variceal development at the right flexure of the colon (Figure 1A-3). Thereafter, the LGV, MCV, and the SRCV arcade were defined as the critical veins [11] (Figure 1A), in which LGV corresponds to the superior route (Figure 1A-1), and the MCV and SRCV arcade correspond to the inferior route (Figure 1A-2,3).

A splenic vein–inferior mesenteric vein (SV-IMV) anastomosis or the preservation of a natural SV-IMV confluence has been emphasized to prevent SPH [7,14,18,19,24,25], whereas some recent reports indicate that inferior mesenteric vein (IMV) preservation was not related to the incidence of SPH [10,11,13,15,17]. Pligrim et al. reported three representative cases of the relationship between gastrointestinal bleeding and the SV-IMV junction. In their report, two patients experienced gastrointestinal bleeding despite the presence of a patent SV-IMV in one patient [19]. Our group and Mizuno et al. suggested that the preservation of the IMV was not associated with SPH in their reports [13,17]. Rosado et al. divided IMV into 15 cases without any occurrence of SPH [15]. As shown in Figure 1D, IMV could be a promising route to bridge the SV to the colonic marginal vein, although it could be replaced by other collaterals, including the arc of Barkow, which connects SV to the colonic marginal vein (Figure 1C). In addition, the IMV sometimes fails to connect with the SMV due to an incomplete colonic venous arcade, which could be one reason as to why the preservation of IMV is not related to SPH occurrence. Thus, IMV is not found to be a critical vein for preventing SPH. However, given that the communication between the arc of Barkow and the colonic marginal vein usually forms postoperatively, there is no guarantee that an adequate connection will develop after surgery; therefore, it may be important to maintain the IMV-SV junction to preserve the collateral route as best as possible if the oncologic goal of the operation can be achieved.

As a result of less superior or inferior routes after PD with PMSC resection, gastrointestinal varices develop in various intestinal regions. Four types of varicose veins were identified: colonic varices, pancreatojejunostomy varices, esophageal varices, and gastrojejunostomy varices [17]. Varicose veins could be created in sites of the abdomen other than those mentioned above (such as rectal varices or varicose veins at the left side of the colon) due to diversity in the collateral route development. Our group reported that the number of remaining critical veins was inversely proportional to the incidence of variceal formation: 0 critical vein, 100% varices, 1 critical vein, 24% varices, 2 or more critical veins, and 0%. Thus, the pathogenesis of SPH is complicated because of the complex hemodynamics of collaterals after PD with PMSC resection.

Some studies indicated no gastrointestinal bleeding even after the incidence of varicose formation [10,14,18,21,26,27,28], but others reported the incidence of severe variceal bleeding [4,5,6,7,8,9,11,13,17,19,24,29,30,31,32,33,34,35,36]. This difference may be due to the dedicated adjustment of SV pressure by collateral routes from the spleen. Since gastrointestinal bleeding cannot occur without variceal formation, the development of gastrointestinal varices is the most important factor for SPH, which is strongly influenced by the number of remaining collaterals draining from the spleen [11,15]. Another important factor for gastrointestinal bleeding is SV pressure. Even after the development of variceal formation, 90% of patients did not experience gastrointestinal bleeding (Table 1). This is because varicose veins alone are not a definitive cause of SPH, and the risk of bleeding seems to be affected by increased SV pressure. The volume of the spleen in patients with SV resection significantly increased 6 or 12 months after surgery compared to before surgery [13,14,16,17,37], and in half of the patients with gastrointestinal bleeding, the spleen volume was doubled or greater compared with preoperative levels [13,17]. Reflecting the increased spleen volume after surgery, the platelet count ratio at 6 months after surgery in the patients after the SV resection was significantly lower than that in patients without the SV resection [13,16,37]. This SV pressure could be controlled by increasing collaterals from the spleen, through methods such as SV reconstruction [12,13,14,15,31,38] or decreasing the blood inflow to the spleen (splenic artery ligation) [16,37].

## 3. Frequency of Variceal Formation and Gastrointestinal Bleeding

Several reports from single centers indicated the incidence of SPH after PD with SV resection, and few reports surveyed the incidence in multiple centers. To investigate the frequency of variceal formation and gastrointestinal bleeding after PD with SV resection/ligation, all the studies that included more than 10 cases of PD with PMSC resection and information of varices and gastrointestinal bleeding are summarized in Table 1 [11,13,14,15,16,17,18,21,31,37]. Technical studies such as “How I do it” were excluded from the analyses. In total, 10 studies were obtained, two of which were multicenter studies [13,37]. Some included overlapping data because they were reported in the same institution or multiple institutions, but were preserved in the table because they had different study concepts or included additional cases or findings. Multidetector enhanced computed tomography was used to detect gastrointestinal varices in all the studies and the endoscopic findings were included in some studies [14,17,18,21].

The frequency of varices ranged from 10.3% to 62.8% [11,13,14,15,16,17,18,21,31,37]. Although some of the authors reported a low incidence of variceal formation [14,15,18], the definition of varicose veins varied from study to study. Most of the studies evaluated esophageal, gastric, pancreatic, and colonic varices; however, a few of the studies did not include pancreatic or colonic varices (Table 1). Hattori and Tanaka H et al. excluded colonic varices from their analysis; consequently, the incidence of varices was relatively low, ranging from 10.3% to 22.6%. Conversely, Rosado et al. [15] evaluated all types of varicose veins but reported a low incidence of varicose veins (20%) in their study, at a 1/3 of the rate reported by our group [17]. To explain this difference, S. M. Strasberg personally contacted A. Saiura and noted, in the discussion [15], that they preserved the whole greater omentum for the left-to-right omental venous channels (Figure 1C), while our group resected the portion of the right side of the greater omentum (Figure 1C-2), which resulted in a high incidence of right colonic varices. Aside from in the studies with a low incidence of varicose veins, the incidence of variceal formation has been reported to range from 37% to 62.8%, but this percentage is highly dependent on the number of preserved collateral veins or other factors, as shown in the subgroup analysis of each report (Table 1).

The rate of gastrointestinal bleeding was approximately 10% in cases of varicose vein formation [11,13,16,17], although Addeo et al. [31] reported a low incidence of gastrointestinal bleeding, for which the dilated collateral veins were included as varices, meaning that they may have overestimated the incidence of variceal formation [31]. Gastrointestinal bleeding was not reported in any of the cases after PD with splenic artery ligation. The reason for this may be that the SV pressure was well-controlled in these cases, and the low SV pressure decreased the risk of gastrointestinal bleeding, even after varicose vein formation. Importantly, bleeding from gastrointestinal varices is categorized as a late-onset postoperative complication. Mizuno et al. summarized 10 cases of bleeding, occurring at a median of 20 months (8–99 months), postoperatively. As a result of the improved prognosis of PDAC through multidisciplinary treatment, more patients may experience SPH in the future.

## 4. Prevention and Prediction of SPH during Surgery

To prevent or reduce the incidence of variceal formation and gastrointestinal bleeding, it is important to preserve the collateral veins wherever possible. Considering the collateral routes after PD with PMSC resection, the LGV, MCV, and SRCV arcades correspond to the critical veins for preventing SPH (Figure 1A), and the incidence of variceal formation could be reduced by preserving these veins [14]. However, it is sometimes difficult to preserve these veins due to a tumor invasion, or to obtain adequate surgical margins. Moreover, even if these veins are preserved during surgery, it is uncertain whether they will remain patent and functional postoperatively. Thus, in addition to these veins, the following potential collaterals should be preserved wherever possible: SV-IMV confluence, omentum, and other retroperitoneum collaterals [15]. As shown in Figure 1D, the IMV is not a direct drainage route from the spleen to the SMV, although it performs an important role in connecting the SV to the colonic marginal vein. When preserving the omentum, Rosado et al. attested to the importance of preserving the spleno-colic omentum because it is the route by which the SV is connected to the greater omentum and the marginal vein of the transverse colon (Figure 1C-1). They also emphasized the importance of preserving the connection of the right side of the colon and omentum to maintain the route from the omental veins to the colonic marginal vein (Figure 1C-2). Retroperitoneum collaterals may contain a shunt that leads from the spleen to systemic circulation and it would be beneficial to preserve them.

Apart from collateral venous preservation, there are several surgical interventions that can prevent SPH during PD. To preserve the confluence of SV, Cusack et al. introduced the now widely applied [6,36] interposition grafting technique [8,9] (Figure 2A). Clavien et al. reported a wedge resection of the PV to preserve SV confluence during PD (Figure 2B). These techniques are applicable only for tumors that partially invade the PV/SMV. A wide resection of the PV is sometimes necessary in cases of pancreatic head and neck cancer displaying PV/SMV invasion, and this may require the transection of the SV. Although extended PD may require a complex surgical technique, it has the advantage of obtaining a wider surgical margin and could improve the survival chances of patients with PDAC with PV invasion [39]. In the case of SV transection, various types of SV reconstruction can be performed [12,24,31,34,40,41] and several articles reported that SV reconstruction could reduce the incidence of SPH [12,31,42]. SV reconstruction with the PV [42,43,44], IMV [7,24] or LRV [34,41] has been previously described. Although SV-PV anastomosis is simple and feasible [12,42] (Figure 2C), this technique is not applicable in the occurrence of a wide gap between the SV and PV, and has a potential risk of PV stenosis due to the distortion at anastomosis [34]. An SV-IMV anastomosis consists of part of an inferior route and is not a critical vein in preventing variceal formation [10,15]. Therefore, the preservation or restoration of the collateral flow into the IMV is not a substantial solution to prevent SPH [19]. In contrast, SV-LRV anastomosis for extended PD is widely applicable and technically feasible [12,34,41] (Figure 2C). Many other techniques for SV reconstruction, including splenogonadal, spleno-left adrenal, and splenojejunal vein or spleno–interposition graft–PV/LRV anastomoses are technically possible, but these techniques require anastomosis with sall-caliber veins and therefore result in a high incidence of anastomotic obstruction [12].

A splenic artery resection (SAR) during PD with PMSC resection is also a potential technique through which to prevent SPH after surgery. Both Gyoten et al. and Yamada et al. successfully reduced the incidence of varices by PD-SAR without increasing the risk of complications such as diabetes mellitus, and furthermore, variceal bleeding was not observed in their study [16,37]. Although SAR may prove to be useful in terms of reducing the SV pressure after PD with PMSC resection, this approach does not alter the number of preserved critical veins. Indeed, variceal formation developed in some patients [13,16,37], and one of our patients experienced gastrointestinal bleeding after PD-SAR (unpublished data). The long-term outcomes for a larger cohort need to be investigated to assess whether this technique is a reasonable means through which to prevent SPH.

For the prediction of SPH during surgery, our group measured the SV pressure during surgery and surveyed the occurrence of SPH after PMSC resection. We reported that, in conditions where the SV pressure after SV clamping measured more than 20 mmHg or the difference in SV pressure before and after SV clamping measured over 10 mmHg, approximately 90% (86% and 91%) of the patients developed SPH. We concluded that a high SV pressure after clamping SV (≥20 mmHg) and a large SV pressure difference (≥10 mmHg) before and after clamping SV act as feasible indication criteria for SV reconstruction to prevent SPH. Yamada et al. [37] also measured SV pressure during surgery and confirmed that clamping the SV increased the SV pressure to more than 28 cmH_2_O and that clamping both the SV and the splenic artery decreased the SV pressure to less than 20 cmH_2_O. They concluded that SA ligation significantly decreased the development of digestive varices without causing clinically significant complications. This result indicates that the incidence of SPH was suppressed by decreasing the SV pressure.

Ultimately, the surgeons’ expertise and advanced surgical skills are critical in avoiding SPH.

## 5. Treatment of Variceal Bleeding

Patients with asymptomatic varicose veins are not eligible for treatment, but repeated bleeding or anemia could prove to be fatal and should be treated. Splenectomy or splenic arterial embolization has been conventionally employed for gastric varices after SV occlusion [45,46,47,48], and recently, SV stenting has also been introduced as a safe and effective treatment for SPH-related gastrointestinal bleeding [49]. Similarly, several treatments combatting gastrointestinal bleeding for SPH after PD with PMSC resection have been reported [11,13,16,17,18,19,29,30,32,33,37]. At the point of determining the appropriate treatment, it is important to recognize the number of developed varicose veins and the location of the bleeding. If there are multiple varicose veins or the bleeding site is unclear, splenectomy or splenic arterial embolization is recommended [11,13,16,17,19,32]. If the varices and the bleeding site are localized interventional radiology, endoscopic, or conservative treatment can be implemented. Either endoscopic variceal ligation or endoscopic hemostasis were applied for esophageal [13,16,17,18], pancreatojejunostomy [16], gastrojejunostomy [37] or colonic varices [16,31]. Interventional radiology, such as the obliteration of the varices via a transhepatic portal venous approach, is also utilized for localized varices and bleeding [29,30,32,33,37]. Some patients experienced repeat bleeding even after treatment. Our group reported that one patient experienced gastrointestinal bleeding even after partial splenic arterial embolization, and therefore required a splenectomy [11]. Gyoten et al. [16] described the case of four patients who developed gastrointestinal bleeding. One patient underwent emergency endoscopic clipping, transarterial embolization, distal gastrectomy, and re-anastomosis of gastrojejunostomy for repeated bleeding; however, the patient died of disseminated intravascular coagulation. Taking into consideration that persistent bleeding might occur due to splenic venous hypertension, inadequate therapy may lead to the requirement of additional treatment or potentially fatal results [16]. Thus, some patients still bleed repeatedly even after conservative treatment, local treatment, or partial splenic embolization [11,16]. It is important to remember that splenectomy might be a useful solution for patients with recurrent bleeding providing that the patient’s condition allows the treatment, and that the cancer recurrence is well-controlled.

## 6. Conclusions

In this review article, we summarized the pathogenesis, frequency, prevention, and treatment of SPH. The pathogenesis of SPH is complicated due to complex collateral routes after PD with a PMSC resection; however, the incidence of variceal formation is reported to range from 37% to 62.8%, and the rate of gastrointestinal bleeding was approximately 10% in cases of varicose vein formation. To prevent SPH, it is important to preserve collateral veins wherever possible, and to understand that SV reconstruction, especially SV-LRV reconstruction, is a useful technique to increase the critical veins for SV drainage. There are several options for the treatment of variceal bleeding, including endoscopic treatment, interventional radiology, splenic arterial embolization, or splenectomy.

The prognosis of PDAC has been gradually improving due to the progress of multidisciplinary therapies, and the risk of SPH after PD with PMSC resection is no longer negligible. We should attempt to prevent SPH during surgery, and if gastrointestinal bleeding occurs after surgery, the optimal treatment should be selected.

## Figures and Tables

**Table 1:** Frequency of variceal formation and gastrointestinal bleeding after PD with SV resection/ligation.

No	Author	Journal	Year	Case No. of SV Resection	*N*	Varices	Type of Evaluated Varices				Bleeding
							Esophageal	Gastric	Pancreatic	Colonic	(% of Varices)
1	Ono et al. [17]	Br. J. Surg.	2015	Total cases (LGV−, MCV−)	43	27 (62.8%)	✔	✔	✔	✔	3 (11.1%)
2	Hattori et al. [18]	Dig. Surg.	2015	Total cases	81	7/31 (22.6%) *	✔	✔	-	-	1 (14.3%)
3	Gyoten et al. [16]	World J. Surg.	2016	Total cases	72	44 (61.1%)	✔	✔	✔	✔	4 (9.0%)
				SAR−	58	39 (67.2%)					4 (10.3%)
				SAR+	14	5 (35.7%)					0
4	Rosado et al. [15]	J. Gastrointest Surg.	2017	Total cases (IMV−)	15	3 (20.0%)	✔	✔	-	✔	0
5	Tanaka H et al. [14]	HPB (Oxford)	2017	Total cases	29	3 (10.3%)	✔	✔	-	-	0
				LGV preservation+	11	0					0
				LGV preservation−	18	3 (16.7%)					0
6	Tanaka M et al. [11]	Surgery	2019	Total cases **	88	41 (46.6%)	✔	✔	✔	✔	5 (12.2%)
				Critical vein: 0	29	29 (100%)					4 (13.8%)
				Critical vein: 1	51	12 (23.5%)					1 (8.3%)
				Critical veins: ≥ 2	8	0					0
7	Mizuno et al. [13]	Ann. Surg.	2019	Total cases	251	93 (37.1%)	✔	✔	✔	✔	10 (11.0%)
				SAR−/SV reconstruction-	227	84 (37.0%)					9 (10.7%)
				SAR+	12	4 (33.3%)					0
				SV reconstruction+	12	5 (41.6%)					1 (20%)
8	Addeo et al. [31]	Surgery	2020	Total cases	114	68 (59.6%)	✔	✔	-	✔	1 (1.5%)
				SV reconstruction-(IMV−)	36	29 (80.6%)					1 (3.4%)
				SV reconstruction+	78	39 (50%)					0
9	Shiihara et al. [21]	Pancreatology	2020	Total cases	36	20 (55.6%)	✔	✔	✔	✔	0
				LGV preserved via PV	8	0					0
				IMV preserved (LGV−)	8	2 (25%)					0
				LGV−, IMV−	20	18 (90%)					0
10	Yamada et al. [37]	Langenbecks Arch. Surg.	2021	Total cases	63	16 (25.4%)	✔	✔	✔	✔	NA ***
				SAR−	21	10 (47.6%)					NA ***
				SAR+	42	6 (14.3%)					0

*: Varices were assessed in 31 patients and detected in 7 patients. **: Patients with patent reconstructed SV were excluded. Critical veins indicate LGV, MCV, and SRCV arcade. ***: Variceal bleeding after PD-PVR without SAR (*n* = 50) occurred in 6 patients (12%), although no description was observed pertaining to the percentage of patients who bled after SV resection. ✔ indicates that the journal has evaluated the varices. #1 and #6, and #2 and #5 were reported by the same institute, respectively. #7: Data were collected from 40 centers in Japan and included some of the patients from #1, 2, 3, 5, 6, and 9. #10: Data were collected from 2 centers in Japan. IMV, inferior mesenteric vein; LGV, left gastric vein; MCV, middle colic vein; NA, not applicable; SAR, splenic artery resection; SV, splenic vein.
